# Avoidant Restrictive Food Intake Disorder in a 28 year old man: a case report

**DOI:** 10.1192/j.eurpsy.2024.1159

**Published:** 2024-08-27

**Authors:** P. Setién Preciados, E. Arroyo Sánchez, C. Díaz Mayoral

**Affiliations:** Servicio de Psiquiatría, Hospital Universitario Príncipe de Asturias, Alcalá de Henares, Spain

## Abstract

**Introduction:**

Avoidant Restrictive Food Intake Disorder is a disorder included among the eating disorders criteria group. Prevalence and incidence rates of ARFID in the general population remain largely unknown. Despite ongoing variability in the interpretation of diagnostic criteria in clinical practice, good progress has been made regarding recognition and assessment of ARFID. Different approaches to treatment are currently being explored, with reported outcomes for ARFID vary, consistent with the heterogeneity of the disorder. At present, there is insufficient evidence to determine the likely course and prognosis.

**Objectives:**

Review what avoidant restrictive food intake disorder consists of, the challenges it presents, as well as its prognosis and potential treatments.

**Methods:**

Presentation of a patient’s case and review of existing literature, in regards to ARFID.

**Results:**

The patient in question is not clear he can be diagnosed of avoidant restrictive food intake disorder given his OCD symptoms, which are intertwined. That said, he does not have body dysmorphophobia and does check for all the ARFID criteria. Their prognosis is not good, having failed several psychological and pharmacological treatments.

In literature, there is not much evidence around the disease because of its novelty, being recently included in the DSM 5 as a new class of eating disorders (EDs), not finding high quality studies (meta analysis, systematic review). ARFID is characterised by a lack of interest in eating or avoiding specific types of foods because of their sensory characteristics. This avoidance results in decreased nutritional intake, eventually causing nutritional deficiencies. In severe cases, ARFID can lead to dependence on oral nutritional suplemments, which interferes with psychosocial functioning. The prevalence of ARFID can be as high as 3% in the general population, and it is often associated with gastrointestinal symptom. Given the high prevalence of ARFID, a rapid and systematic nutrition survey should be conducted during every consultation. Its treatment should also be adapted depending on the severity of the nutritional problem and may involve hospitalisation with multidisciplinary care (paediatrician, nutritional therapist, dietitian, psychologists, and speech therapists).

In regards to potential treatments, there is no evidence-based psychological treatment suitable for all forms of ARFID at this time. Several groups are currently evaluating the efficacy of new psychological treatments for ARFID, particularly, family-based and cognitive-behavioural approaches, but results have not yet been published.

**Conclusions:**

Future directions for research could be usefully informed by closer collaboration with other fields, including feeding disorders, emotion processing and regulation, neurodevelopment, and appetite.

**Disclosure of Interest:**

None Declared

